# Revisiting the Impact of Local Leptin Signaling in Folliculogenesis and Oocyte Maturation in Obese Mothers

**DOI:** 10.3390/ijms22084270

**Published:** 2021-04-20

**Authors:** Karolina Wołodko, Juan Castillo-Fernandez, Gavin Kelsey, António Galvão

**Affiliations:** 1 Department of Reproductive Immunology and Pathology, Institute of Animal Reproduction and Food Research of PAS, Tuwima 10, 10-748 Olsztyn, Poland; k.wolodko@pan.olsztyn.pl; 2 Epigenetics Programme, Babraham Institute, Cambridge CB22 3AT, UK; Juan.Castillo-Fernandez@babraham.ac.uk (J.C.-F.); gavin.kelsey@babraham.ac.uk (G.K.); 3 Centre for Trophoblast Research, University of Cambridge, Cambridge CB2 3EG, UK

**Keywords:** leptin, obesity, ovary, folliculogenesis, oocyte

## Abstract

The complex nature of folliculogenesis regulation accounts for its susceptibility to maternal physiological fitness. In obese mothers, progressive expansion of adipose tissue culminates with severe hyperestrogenism and hyperleptinemia with detrimental effects for ovarian performance. Indeed, maternal obesity is associated with the establishment of ovarian leptin resistance. This review summarizes current knowledge on potential effects of impaired leptin signaling throughout folliculogenesis and oocyte developmental competence in mice and women.

## 1. Introduction

Obesity is a prevalent disease worldwide, usually associated with infertility. Studies in obese infertile females show the occurrence of systemic hyperestrogenemia, hyperinsulinemia, and associated ovarian dysfunction through premature follicular atresia and anovulation [1]. Indeed, the ovaries of obese mothers have been shown to accumulate lipids, high levels of reactive oxygen species [2], and inflammatory mediators [3]. Furthermore, obesity has been shown to hamper not only oocyte maturation and quality [4] but also embryo development [5], with reported long term effects and direct causality between obesity in the mother and prevalence of cardiovascular disease or cancer in the offspring.

We have recently identified striking links between gain in maternal body weight and global gene expression profile in cumulus cells [6], reiterating not only the importance of maternal metabolic state for ovarian function but also the dynamics of temporal alterations in the ovary throughout obesity progression [6]. In the same work, we demonstrated the establishment of leptin resistance in the ovary of diet-induced obese (DIO; abbreviations listed after the main text) mice after 16 weeks (wk) in comparison to 4 wk [6]. Hence, increased levels of leptin signaling inhibitor suppressor of cytokine signaling (SOCS) 3 were observed already after 4 wk DIO [6] with potential implications for ovarian pathogenesis during early obesity.

In the present narrative review, we revisit, first, physiological aspects governing folliculogenesis in mice and women, discussing the major molecular pathways preconizing developmental changes in both germ line and somatic cells. Second, we consider how maternal obesity and, particularly, local changes in leptin signaling can affect the regulation of such transitions, debating potential outcomes for oocyte quality and early embryo development.

## 2. Obesity and Ovarian Function

The worldwide epidemic of obesity has reached unprecedented levels and infertility is described as an associated comorbidity [1]. Indeed, obese women were linked to poor reproductive outcomes, such as anovulation or decreased conception rate [7]. Literature has evidenced the link between peripheral insulin resistance and functional hyperandrogenism and hyperestrogenism [8], the main causes of anovulation and reduced endometrial receptivity [1]. Studies in mice have shown how obesity-associated hyperinsulinemia and diabetes can lead to delayed oocyte maturation in preovulatory follicles and apoptosis in granulosa cells (GC) [9]. Furthermore, ovaries of obese women were shown to present high levels of androstenedione and testosterone [8], responsible for premature follicular atresia. Therefore, the endocrine imbalance observed in obese mothers affects directly the follicular pool, reducing the number of primordial follicles and compromising fertility [10].

In recent years, studies, particularly in mouse models for obesity, have clearly revealed the main readouts in ovaries from obese mothers. Indeed, the accumulation of lipids in oocyte and surrounding GC or cumulus cells (CC) was shown to culminate with lipotoxicity and inflammation, GC apoptosis, endoplasmic reticulum (EnR) stress in cumulus–oocyte complexes (COCs) [11], and oocyte mitochondrial dysfunction [12]. Furthermore, we have also shown, through DIO protocols in mice, dramatic changes in the expression of genes regulating cytoskeletal organization and the formation of transzonal projections (TZPs), which play a key role mediating the crosstalk between somatic cells and oocyte during follicular growth [6]. Thus, maternal obesity has been linked to changes in local cytokine milieu and signaling mediators of follicle growth, maturation, steroidogenesis, and ovulation [3]. Ultimately, the oocyte quality was shown to be equally impaired, with delayed meiotic maturation, abnormal mitochondrial distribution, and oxidative stress [13]. Finally, Robker and collaborators reported altered follicular fluid levels of metabolites such as C-reactive protein and androgen activity in women [14], which have been associated with cellular stress and impaired oocyte nuclear maturation [4]. Undeniably, literature comprehensively describes the main features in the ovaries of obese mothers, but fewer studies have functionally addressed the mounting response leading to ovarian failure and hampered oocyte quality. Noteworthy, describing temporally the progression of events in the ovaries of obese mothers is fundamental to understanding the pathophysiology of ovarian failure and helping us delineate adequate treatments regarding disease progression.

### 2.1. Leptin—A Common Denominator between Ovarian Function and Obesity

During obesity progression, the ever-growing adipose tissue secretes large amounts of leptin, which causes systemic hormonal imbalance. Leptin is mainly known to regulate appetite at the central level [15] besides modulating the release of gonadotropin releasing hormone (GnRH) neuron activity and gonadotropins [16]. Nonetheless, leptin is also an important modulator of ovarian function [17]. Leptin long and short receptor isoforms were detected in most cell types in murine ovary, particularly in the oocyte [17]. Likewise, both isoforms of the leptin receptor (ObR) were previously detected in human GC and theca cells (TC) [18]; as well, leptin and leptin soluble receptor were detected in human follicular fluid [18,19]. Thus, leptin signaling components’ heavy representation in the ovary makes the organ particularly vulnerable to the systemic hyperleptinemia observed in obese mothers [20].

**Figure 1:**
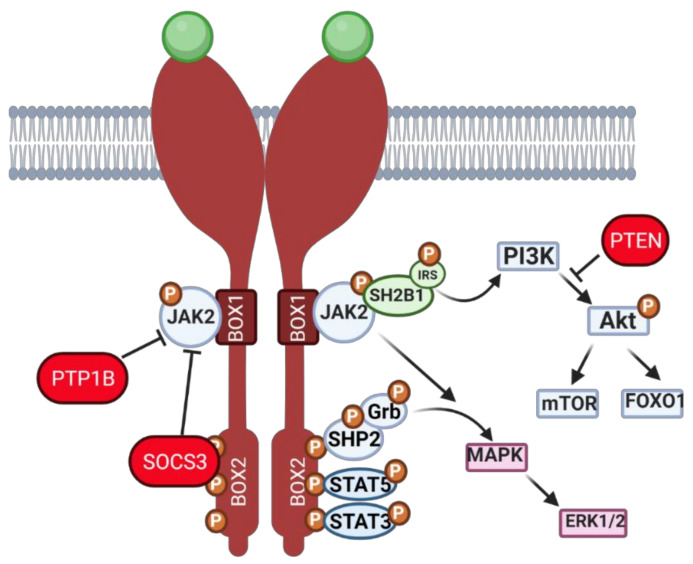
Schematic representation of leptin signaling pathway cascade. Leptin binds to its dimerized membrane receptor, and signal propagation starts. Janus kinase 2 (JAK2) phosphorylation causes transition of phosphate groups to three tyrosines within BOX2 of ObR and activation of (i) SH2-domain containing protein tyrosine phosphatase (SHP-2), which, in turns, binds its adapter molecule Grb-2 and activates downstream signaling, resulting in extracellular signal regulated kinase (ERK) 1/2 activation, (ii) signal transducer and activator of transcription (STAT) 5 activation and (iii) *STAT3* activation. The phosphorylation of JAK2 also activates the MAPK signaling pathway and promotes SH2B adaptor protein 1 (SH2B1) and insulin receptor substrate (IRS) binding, which initiates the phosphatidylinositol 3 kinase (PI3K) pathway, which leads to phosphorylation of protein kinase B (Akt), mammalian target of rapamycin (mTOR) and forkhead box O1 (FOXO1) activation. During hyperactivation of the leptin signaling pathway, two main inhibitors can be transcribed—protein tyrosine phosphatase (*PTP*) 1B, which dephosphorylates JAK2 and suppressor of cytokine signaling (SOCS) 3, which blocks tyrosine and JAK2 phosphorylation. Created with BioRender.com, accessed on 1 November 2020.

When leptin signals through the long isoform of its membrane receptor, leptin receptor b (ObRb), a canonical cascade activates Janus kinase (JAK)/signal transducer and activator of transcription (STAT) signaling. Conversely, noncanonical signaling results in insulin receptor substrate (IRS)/phosphatidylinositol 3 kinase (PI3K)/protein kinase B (Akt) and mitogen-activated protein kinase (MAPK)/extracellular signal regulated kinase (ERK) signaling pathways activation (Figure 1). After binding the dimerized ObRb, leptin initially mediates the phosphorylation of JAK2 with further transfer of the phosphate group to three tyrosines within the BOX2 of ObRb. As a result: (i) SH2-domain containing protein tyrosine phosphatase (SHP-2) can be phosphorylated, subsequently binding to the adapter Grb-2 and activating downstream ERK1/2; (ii) STAT5 can be phosphorylated; and/or (iii) *STAT3* can be phosphorylated (Figure 1). Intriguingly, leptin can noncanonically regulate components of the insulin signaling cascade. After ObRb activation, IRSs can be phosphorylated by JAK2 with subsequent activation of PI3K pathway. As a result, phosphatidylinositol-3,4,5-triphosphate (PIP_3_) is generated with further activation of PIP_3_-dependent serine/threonine kinases, such as phosphoinositide-dependent kinase (PDK) 1,2, a conserved activator of Akt, which in turn regulates downstream signaling [21]. With regard to the ERK signaling, leptin can activate the MAPK pathway mainly through two different ways: (i) first, after phosphorylation of ObRb tyrosine 985 by JAK2, SHP-2 is recruited and binds Grb-2, resulting in ERK activation [22]; or (ii) second, the short isoform of ObR, which possesses only BOX1 in the receptor domain and lacks tyrosine sites, can also activate SHP-2 and Grb-2 complex through JAK2 (Figure 1), followed by mitogen-activated protein kinase kinase (MEK) 1 and downstream phosphorylation of ERK1/2 [23]. It is noteworthy that, despite its effects on ERK activation, leptin can also interact with protein kinase C (PKC) and further activate ERK, playing both stimulatory and inhibitory effects on PKC.

**Figure 2:**
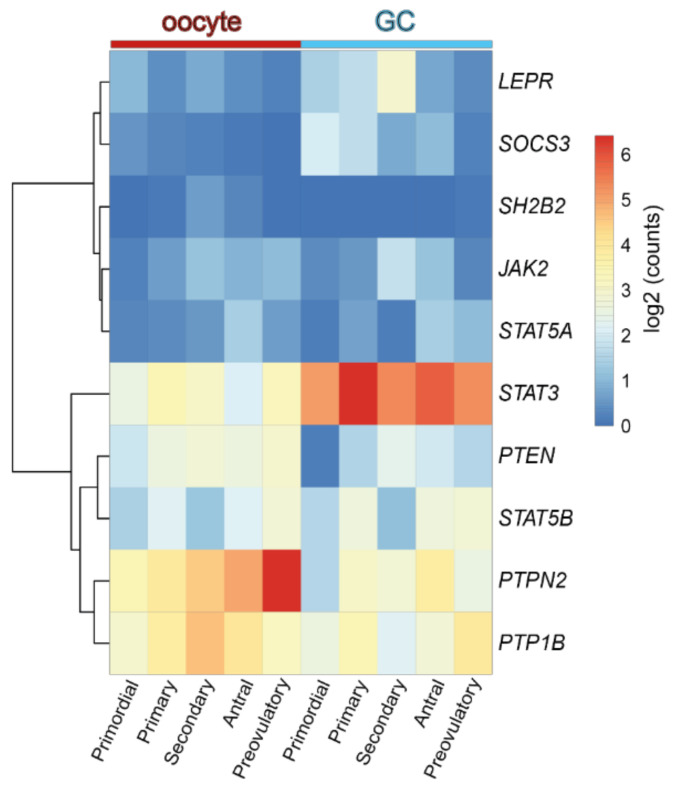
Heatmap representing the expression level of transcripts from the leptin signaling pathway components in human oocyte and granulosa cells (GC) throughout folliculogenesis. Primordial = primordial follicle; Primary = primary follicle; Secondary = secondary follicle; Antral = antral follicle; Preovulatory = preovulatory follicle. Color code from blue to red indicates the relative gene expression level from low to high, respectively. Data from Zhang et al. 2018 [30]. Leptin receptor (LEPR), suppressor of cytokine signaling 3 (*SOCS3*), SH2B Adaptor Protein 1 (SH2B1), Janus kinase 2 (JAK2), signal transducer and activator of transcription (STAT), protein tyrosine phosphatase non-receptor type 2 (*PTPN2*), protein tyrosine phosphatase (*PTP*) 1B.

Concerning the role of leptin in the ovary, evidence gathered from studies with leptin or ObR deficient mice, as well as women, confirmed their infertility and altered pubertal development [24,25]. Functionally, leptin actions in the ovary were shown to have a bimodal nature. In vivo and in vitro studies in mouse ovarian explants [26] evidenced the dose-dependent effect of leptin on progesterone (P4) synthesis, with low doses stimulating and high doses inhibiting expression of enzymes involved in P4 synthesis [26]. Also, studies in other species corroborated the aforementioned observations as the in vitro treatment of equine luteal cells with lower doses of leptin supported P4 secretion, whereas higher doses presented no effect [27]. Particularly regarding follicular dynamics, studies in mice showed that high levels of circulating leptin blocked folliculogenesis, but lower circulating levels of leptin supported the transition from primary to secondary follicle [28]. Conversely, the in vitro treatment of mouse follicles with mouse recombinant leptin once more revealed a dose-dependent response with higher treatment doses inhibiting follicular growth [29]. Presently, we used the data from Zhang and co-workers’ recent report profiling the transcriptome of GC and oocytes isolated from different stages of folliculogenesis in women [30] and plotted the main components of the leptin signaling pathway in order to understand if their expression profile could be linked to a leptin concerted role during particular developmental stages. Indeed, we confirmed some leptin signaling components, such as *protein tyrosine phosphatase non-receptor type 2 (PTPN2), protein tyrosine phosphatase (PTP) 1B*, or *STAT3*, were abundantly transcribed in both oocytes and GCs throughout folliculogenesis, whereas others, such as *SOCS3*, presented very low expression in both cell types under physiological conditions, particularly in later stages of folliculogenesis (Figure 2). Finally, we have recently shown that obesity progression in DIO mice alters leptin signaling in the ovary with increased leptin signaling in the ovary of 4 wk DIO mice, being followed by the establishment of leptin resistance in the ovaries of 16 wk DIO mice [6]. Indeed, expression levels of *SOCS3* in ovarian extracts were dramatically increased already at 4 wk DIO [6]. Therefore, our findings, as well as the observations on leptin signaling component expression in oocytes and GCs from women’s follicles, clearly suggest that the impairment of leptin signaling in the ovaries of obese mothers may contribute to pathogenesis of ovarian failure, particularly given leptin’s established role in ovarian function.

### 2.2. Other Adipokines and Ovarian Function during Obesity

Obesity and progressive expansion of white adipose tissue are largely associated with dramatic changes in the adipokine secretory profile, which ultimately lead to the establishment of a proinflammatory, atherogenic, and diabetogenic systemic environment. Indeed, during obesity, the adipose tissue secretes adipokines other than leptin with potential consequences for ovarian function regulation. Making a full characterization of all adipokines and their potential involvement in ovarian function regulation is certainly beyond the scope of the present review. Nonetheless, literature highlights both local and central regulatory roles of major adipokines, such as adiponectin, visfatin, omentin, and resistin in folliculogenesis [31]. Therefore, in the present section, we briefly discuss potential outcomes for folliculogenesis associated with changes in the secretory profile of main adipokines other than leptin during obesity.

Adiponectin is known as the most abundant circulating adipokine in humans, and its receptors, adiponectin receptor 1 and 2, were shown to be ubiquitously expressed in female reproductive tissues, including the ovaries [32]. Adiponectin’s main systemic roles encompass the increase in insulin sensitivity in both liver and muscle, suppression of hepatic gluconeogenesis, and promotion of fatty acid β-oxidation in the skeletal muscle [33]. Furthermore, adiponectin is also known to have a ‘beneficial’ role in reproduction [31]. Indeed, under physiological levels, a number of adipokines, such as visfatin, omentin and vaspin, or leptin itself, have been described as ‘beneficial’ for reproduction and ovarian function [31]. In this regard, leptin’s prominent role in ovarian function was mainly evidenced by reports on anorectic or undernourished women [34], corroborating the importance of leptin’s exact physiological amounts for ovarian homeostasis [34]. At physiological levels, adiponectin was shown to modulate steroidogenesis and promote oocyte maturation [35], as well as participating in ovulation and supporting early embryo development in various species [35,36]. Interestingly, a recent study has revealed adiponectin’s role in preventing the hyperactivation of primordial follicles in mice treated with a high protein diet [37]. Therefore, decreased circulating levels of adiponectin observed in obese mothers might negatively contribute to ovarian function regulation.

Another particular adipokine, resistin, was shown not only to be increased in circulation during obesity, but also to synergize with leptin on *SOCS3* upregulation in various tissues [38]. Centrally, resistin was shown to lower hypothalamic ObRb transcript levels, contributing to central leptin resistance [38]. Indeed, resistin’s role modulating the secretion of reproductive hormones at the pituitary level was previously evidenced in sheep [39]. As revisited elsewhere, resistin was shown to be widely expressed in the ovaries, particularly in rodent, bovine, porcine, and human TC, GC, and oocytes, modulating steroidogenesis and supporting androgen production [40]. Hence, resistin appears to be another major adipokine capable of modulating ovarian function in obese mothers. Therefore, the multiple contribution of different adipokines to ovarian failure during obesity shall not be neglected. Indeed, the difficulties of studying obesity and its comorbidities rely on its polygenic nature, very often largely simplified by the use of monogenic disease models.

## 3. Revisiting Folliculogenesis in Mice and Women: A Morphofunctional Characterization

**Figure 3:**
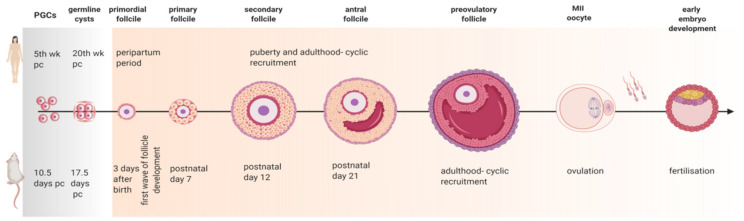
Diagram of folliculogenesis in mice and women. Approximately five weeks (wk) post coitum (pc) in women and 10.5 days (d) pc in mice, primordial germ cells (PGC) arrive at the genital ridge. Germline cysts start breaking 15 wk later in women and 7 days later in mice, creating the primordial follicles. During the peripartum period in woman, and within 3 days after birth in mice, primordial follicles are created. The process of follicle development in women is asynchronous, with menstrual cyclicity being started at puberty. In mice, the first wave of follicle development is detected around postnatal day 7, when the first primary follicles are originated, followed by secondary follicles detected at postnatal day 12 and early antral follicles around postnatal day 21. When mice reach sexual maturity, cyclic recruitment of follicles begins. After ovulation metaphase II (MII) oocyte is released into the oviduct where fertilization takes place, followed by early embryo development. Created with BioRender.com, accessed on 1 November 2020.

The complex nature of folliculogenesis regulation certainly accounts for the vulnerability the ovaries present to the hormonal imbalance seen in obese mothers [7]. Generally, obesity may affect the oocyte and somatic cells at each single developmental stage of folliculogenesis (Figure 3) with the incidence of obesity earlier in life posing a greater threat for the quality of the gamete later in adulthood [41]. Hence, in this section, we revisit folliculogenesis, analyzing major cellular events taking place throughout the long journey the female gamete makes from primordial follicle until fertilization, based on lessons learnt from studies in mice and in humans (Table S1 summarizes the nature of the study presented in the text).

### 3.1. Primordial Follicle Assembly

In the mouse, primordial germ cells (PGC) arrive at the genital ridge on 10.5 days post coitum (dpc) and divide by mitosis with incomplete cytokinesis for the subsequent 3 days, originating the germline cysts (Figure 3) [42]. Next, oogonias enclosed in germline cysts enter the meiosis prophase and are named as oocytes. This is the beginning of a journey with two distinctive ends, the success of ovulation and eventual fertilization or the inevitable condemnation of follicular atresia. Oocyte progresses to the diplotene stage of prophase I of meiosis, remains arrested at this stage from 17.5 dpc, and resumes meiosis only after the surge of luteinizing hormone (LH) just before ovulation (Figure 3) [43]. Prior to cyst breakdown, mitochondria divide and reorganize in the cysts, suggesting a process of active mitochondrial selection [44]. Germline cyst breakdown starts at 17.5 dpc in the mouse [45], and follicles begin to form. The process of primordial follicle assembly is independent from gonadotropin action, being mainly regulated locally by factors like neurotrophin (NT) 4, brain-derived nerve factor (BDNF), folliculogenesis-specific basic helix–loop–helix (FIG-α). Within three days after birth, the cortex of the ovaries is replenished with primordial follicles, consisting of small oocytes surrounded by flattened GC, which remain dormant until awakened by local factors (Figure 3) [44]. In humans, follicular development starts during fetal life, and PGC first reach the gonadal ridge around the fifth wk of pregnancy [46]. They then divide mitotically until the fifth month of pregnancy, when PGC undergo the first meiotic division and become arrested in prophase I. At this stage, germ cells are surrounded by somatic cells, forming the primordial follicles (Figure 3) [46]. In primates, the first wave of primordial follicle activation starts during fetal development, and multiple preantral follicles can be found in the ovary at the sixth month of pregnancy in women, with antral follicles often developing during the next two months [47]. As a result, the ovary of a newborn is replenished with large antral follicles, which will invariably undergo atresia [47].

### 3.2. Primary Follicle Development and Growth

The process of early folliculogenesis encompasses important structural and molecular changes, with GC becoming cuboidal and the oocyte accumulating ribonuclease acid (RNA) and protein. Moreover, stromal/mesenchymal cells associated with the primordial follicle are presumably early stage precursors of TC in the primary follicle [48]. At last, primary follicles are enclosed in basal lamina with one layer of GC (Figure 3). In secondary follicles, fibroblast-like cells from ovarian stroma form the TC layer that surrounds the follicle and, together with GC-derived aromatase, mediates the synthesis of steroid hormones essential for oocyte growth. Follicles then develop a fluid-filled antrum, being designated as antral follicles (Figure 3). Prior to ovulation, the surge of gonadotropins determines the resumption of oocyte meiosis, which progresses from prophase I to metaphase II (MII). Indeed, meiotic division is finished exclusively after fertilization [49]. In women, primordial follicles are formed during the peripartum period with around one to two million follicles being present at birth. These follicles form the follicular reserve, which determines reproductive potential throughout their lifespan. In women, it takes approximately one year for a primordial follicle to mature and reach the ovulation stage. The transition from resting pool into growing follicle is highly dependent on the game of forces between growth/differentiation and pro-apoptotic factors [46].

### 3.3. The Road to Ovulation

As in rodents, the progression from preantral to antral follicles in women determines the transition from gonadotropin-independent to gonadotropin-dependent follicular growth, with further selection of the dominant follicle prior to ovulation. At puberty, centrally released gonadotropins follicle-stimulating hormone (FSH) and LH promote the development of antral follicles and the onset of ovulation, with only one single follicle reaching the preovulatory stage during a menstrual cycle in humans (Figure 3) [46]. Conversely, in cycling mice, few follicles reach the preovulatory stage [50]. In women, approximately seven million oocytes initially start developing in the ovaries, with only two million oocytes present at birth [44] and approximately four hundred ovulating throughout reproductive lifespan [51]. Conversely, the mouse ovaries present great variation in oocyte number between strains, as well as rates of follicle loss [52], with approximately seven thousand oocytes being present in the ovaries at 3 days after birth [53]. Follicular response to the gonadotropin surge results in the rupture of the follicle wall. The dramatic expansion of the follicle volume is mainly associated with increased ovarian blood flow and enhanced capillary permeability, mediated by the activity of local proteolytic enzymes, steroids, and arachidonic acid metabolites [54]. After ovulation, COCs are released into the oviduct where, in the presence of sperm, fertilization takes place. Immediately after fertilization, meiosis is completed, followed by the zygotic first mitotic division and activation of transcription as the embryo develops until the blastocyst stage (Figure 3), the time of implantation in both mice and humans [55].

## 4. Molecular Mechanisms Regulating Primordial to Primary Follicle Transition

The rate of primordial follicle assembly and transition to primary follicle sets both the size and depletion rate of the primordial follicle pool. Therefore, its inadequate regulation culminates with premature follicle loss and sterility. The formation of the follicular pool and the first wave of primordial follicle activation is a synchronous process in rodents and takes place between days 3 and 7 postnatal (Figure 3). Nonetheless, due to the lack of gonadotropin activity, these follicles inevitably undergo atresia [56]. In primates, the process is asynchronous with some primordial follicles leaving the resting pool before others start meiosis [56], whereas remaining germ cells, mainly located peripherally, still undergo mitosis [53]. Asynchronous follicle activation in domestic animals and humans is notoriously difficult to study, hence, most of the studies on molecular regulation of primordial follicle activation were undertaken in rodents. In the present section, we discuss the molecular mechanisms controlling primordial to primary follicle transition, and how obesity and altered leptin signaling can jeopardize its regulation.

### 4.1. The PI3K Pathway and Primordial to Primary Follicle Transition

**Figure 4:**
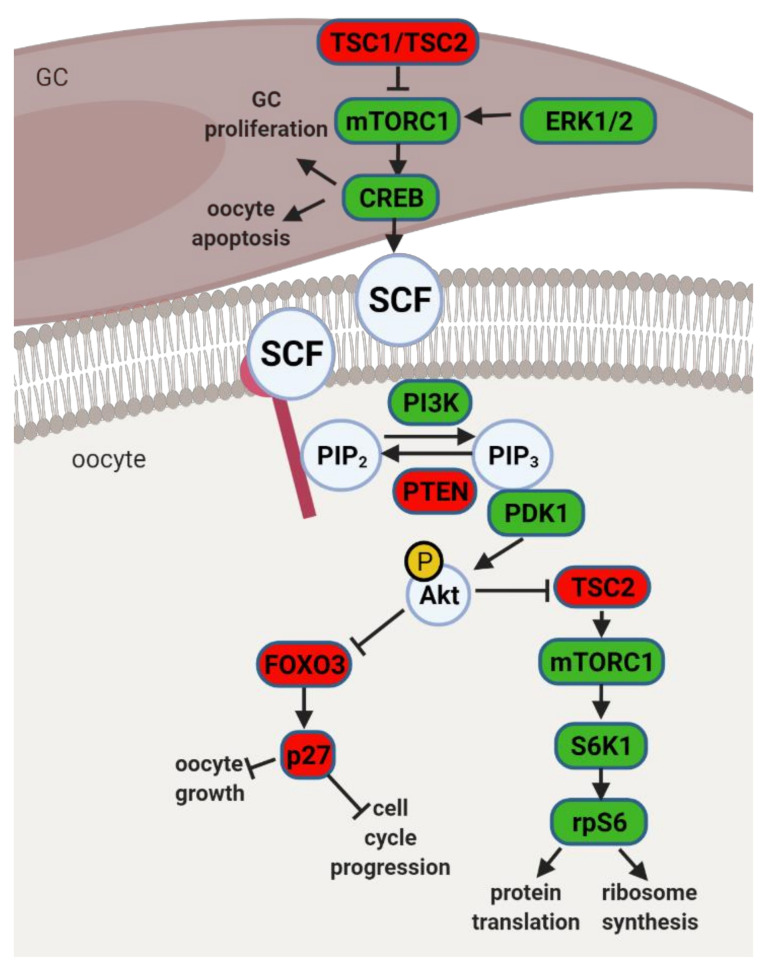
Schematic representation of mammalian target of rapamycin (mTOR) and phosphatidylinositol 3 kinase (PI3K) signaling pathway and its downstream regulators in the granulosa cells (GC) and oocyte. Extracellular signal-regulated protein kinase ½ (ERK1/2) activates mTOR complex 1 (mTORC1) in pre-GC to initiate the activation of primordial follicles. mTORC1 activates cyclic AMP-response element binding protein (CREB), which promotes stem cell factor (SCF) transcription and stimulates PI3K signaling but also affects pre-GC proliferation and oocyte apoptosis. mTOR signaling negative regulators TSC1 and TSC2 suppress mTORC1 activity. PI3K signaling pathway is activated by SCF in the oocyte. PI3K phosphorylates phosphatidylinositol-4,5-biphosphate (PIP_2_) to phosphatidylinositol-3,4,5-triphosphate (PIP_3_), which interacts with 3-phosphoinositide dependent protein kinase 1 (PDK1) for subsequent phosphorylation of protein kinase B (Akt) and forkhead box O3 (FOXO3) with its downstream mediator, cyclin-dependent kinase inhibitor 1B (p27). Upon phosphorylation, the inhibitory effect of FOXO3 and p27 on cell cycle progression and oocyte growth is inhibited, and the primordial follicle is recruited. Akt also activates ribosomal protein S6 kinase beta-1 (S6K1) through inhibition of TSC2 with subsequent mTORC1 activation and further ribosomal protein S6 (rpS6) phosphorylation, which leads to protein translation and ribosome synthesis. Factors indicated in red are associated with follicle dormancy; molecules indicated in green are associated with follicle activation. Created with BioRender.com, accessed on 1 November 2020.

Mouse studies with three particular phenotypes of transgenic animals unraveled the main molecular pathways involved in primordial follicle activation. The first phenotype consisted of mice with premature follicle activation, follicle loss, and sterility. The second phenotype presented follicles arrested in the primordial follicle stage, and in the third phenotype, follicles did not progress beyond the primary follicle stage. Studies on aforementioned models identified the PI3K pathway in the oocyte, together with the mammalian target of rapamycin (mTOR) pathway in pre-GC, as major regulators of primordial follicle activation (Figure 4). Indeed, ERK1/2 may regulate mTOR Complex (mTORC) 1 in pre-GC with subsequent activation of cyclic adenosine monophosphate (cAMP)-response element binding protein (CREB), which is known to promote the transcription of stem cell factor (SCF), a main activator of PI3K signaling in the oocyte [57]. Also, studies with CREB conditional knockdown in the ovary showed its regulatory role on pre-GC proliferation and oocyte apoptosis [57]. After PI3K activation in the oocyte, phosphatidylinositol-4,5-biphosphate (PIP_2_) is phosphorylated into PIP_3_, followed by Akt phosphorylation by PDK1. In fact, Akt may further interact with forkhead box O3 (FOXO3) or ribosomal protein S6 kinase beta-1 (S6K1) (Figure 4). Upon phosphorylation, pFOXO3 translocates from the nucleus to the cytoplasm, thereby losing transcriptional activity. Indeed, FOXO3 works as a molecular switch, ensuring, together with cyclin-dependent kinase inhibitor 1B (p27), follicle dormancy, and blocking follicular recruitment and oocyte growth [58]. Alternatively, Akt may also phosphorylate and inhibit tuberous sclerosis complex (TSC) 2, allowing for the activation of mTORC1 and S6K1/ribosomal protein S6 (rpS6), a pathway mainly responsible for protein translation and ribosome synthesis (Figure 4) [59]. Thus, Akt plays a key role in the primordial follicle fate, mediating survival during dormancy, as well as follicle activation or atresia [59].

Studies in bovines and baboons have linked PDK1 deficiency, and subsequent downregulation of rpS6 in oocytes, to premature ovarian failure (POF) and loss of primordial follicles [59]. On the other hand, deficiency of phosphatase and tensin homolog (PTEN), a PI3K inhibitor, was seemingly associated with the overexpression of rpS6 and POF due to hyperactivation of primordial follicles and consequent follicular atresia [59]. Mutations in PTEN suggested its importance for maintenance of the primordial follicle pool [60]. Indeed, PTEN, together with regulators TSC1 and TSC2, negative regulators of mTOR signaling, maintain the quiescence of primordial follicles [61]. Furthermore, in vitro treatment of human ovarian cortical tissue with PTEN inhibitor resulted in primordial follicle development to preovulatory stage [62]. Finally, studies with porcine neonatal ovaries also showed the localization of PTEN, Akt, and FOXO3 in oocytes and GC of primordial, primary, and secondary follicles, suggesting their role in primordial follicle activation across species [63]. Literature describes additional factors involved in PI3K regulation and primordial follicle activation, particularly, growth differentiation factor (GDF) 9 [64] or SCF receptor (Kit). Mutations of the aforementioned factors resulted in folliculogenesis arrest at the primary follicle stage [65]. Finally, the involvement of insulin was also reported in primordial to primary follicle transition and its ability to inhibit FOXO3 through Akt signaling [66]. Taken together, PI3K and mTOR signaling pathways appear to be master regulators of primordial follicle activation. Precise control of these pathways is essential for maintenance of the female reproductive lifespan and the preservation of primordial follicles in quiescence.

Although the molecular mechanisms driving primordial to primary follicle transition in women remain much less clear than in the mouse, a recent study by Zhang et al. identified a number of genes differentially expressed at this stage [30]. As previously discussed in Section 2.1, the leptin signaling inhibitor *SOCS3* presented its highest expression level in GC from primordial follicles (Figure 2). Furthermore, functional pathways associated with differently expressed genes (DEGs) during primordial to primary transition included insulin signaling, GnRH, NT, and mTOR–PI3K, JAK–STAT pathways in both oocytes and GC [30]. Thus, components of leptin signaling appear to be amid the physiological factors regulating primordial to primary follicle transition in humans.

In conclusion, studies in transgenic mice with oocyte and GC specific mutations helped us identify the major roles of PI3K and mTOR signaling mediating primordial follicle activation. More recently, a study in humans has revealed the involvement of JAK–STAT signaling and *SOCS3* in early folliculogenesis, highlighting the importance of the leptin signaling pathway in primordial to primary follicle transition.

### 4.2. Obesity, Leptin Signaling and Primordial Follicle Activation

Studies in rodents have highlighted the effects of maternal obesity on ovarian failure and, particularly, on PI3K dysregulation. A report in rats showed that DIO treatment led to POF through activation of mTOR and suppression of sirtuin (SIRT) 1 signaling [67], whereas the ovaries of mice fed a high-fat diet (HFD) presented aberrant expression levels of PI3K pathway components [68]. Indeed, the putative crosstalk between leptin and the PI3K pathway can be anticipated, mainly, through components of the insulin signaling cascade (Figure 1). After ObRb activation, JAK2 can phosphorylate IRs, with subsequent activation of PI3K pathway [21]. This generates PIP_3_, which further activates PIP_3_-dependent serine/threonine kinases, such as PDK1,2, responsible for activation of Akt, as previously discussed (Figure 1) [21]. Indeed, a study in mice with a triple mutation in ObR tyrosines linked follicle loss to the activation of PTEN/PI3K/Akt/mTOR signaling [69]. Furthermore, Panwar and colleagues showed the direct effects of leptin on the follicular pool once passive immunization of prepubertal mice against leptin prompted the transition of primordial to primary follicles [28]. Therefore, adequate levels of leptin signaling in the ovary seem to prevent primordial follicle hyperactivation and help to maintain the follicular pool. Concordantly, these observations suggest the decrease in leptin signaling we have reported in the ovaries of 16 wk DIO mice [6] might accelerate the activation and depletion of the primordial follicle pool.

We have previously linked *SOCS3* overexpression in ovarian extracts of 16 wk DIO mice with the establishment of leptin resistance [6]. Despite no previous characterization of *SOCS3*’s putative role on follicular pool activation, its ability to inhibit the phosphorylation of JAK2 and ERK is well established. As a result, SCF signaling could be disturbed in GC (Figure 4), as well as PI3K activation in the oocyte, during primordial to primary follicle transition in obese mothers. After reanalyzing Zhang’s data, we plotted the expression level of leptin signaling components across all stages of folliculogenesis (Figure 2). Indeed, we confirmed *SOCS3* mRNA levels remained very low in both oocyte and GC throughout folliculogenesis in women with regard to other components of the pathway (Figure 2). Therefore, our previous results showing a dramatic increase in *SOCS3* protein and mRNA levels in the ovaries of 16 wk DIO mice invite the speculation of PI3K putative dysregulation in primordial oocytes. Finally, the high mRNA level of *PTPN2* observed in the oocyte throughout folliculogenesis was also of particular relevance [30]. In fact, *PTPN2* has also been associated with the establishment of leptin resistance at central level [70] and, therefore, appears to be an important candidate in the pathophysiology of ovarian failure in obese mothers.

To conclude, previous associations between maternal obesity and decreased follicular count can be linked to changes in local leptin signaling. Effects of leptin resistance can be measured not only by lack of leptin action maintaining the follicular pool but also through excessive levels of *SOCS3*, potential dysregulation of PI3K signaling, and accelerated primordial follicle activation.

## 5. Molecular Regulation of Early Antral to Preovulatory Follicle Transition

The regulation of primary to secondary follicle transition is a process mostly paracrinally regulated that relies mainly on the communication between GC and oocyte. Morphologically, GC proliferate from a single monolayer to multiple layers with rapid expansion of the oocyte. Molecularly, the process is coordinated by local factors such as GC-derived anti-Mullerian hormone (AMH) [71], which was shown to regulate oocyte-derived GDF9 activity in follicular growth [64]. In addition, the factors Kit and SCF [65], as well as LIM homeobox protein (Lhx) 8, were shown to be involved in primary to secondary follicle transition [72]. Finally, NOTCH [73] and mTOR signaling were reported to control GC proliferation in primary follicle growth [74]. Conversely, after antrum formation, the follicles then become responsive to gonadotropins, responsible for orchestrating major events leading to preovulatory follicle formation, such as steroidogenesis, intercellular communication, GC differentiation, and oocyte expansion. In this chapter, we discuss the main pathways regulating preovulatory follicle formation, the particular involvement of leptin signaling, and the disruptive effects of obesity and impaired leptin signaling.

### 5.1. Preovulatory Follicle Formation—The Role of Estradiol

The systemic increase in pituitary-released FSH was shown to promote growth of early antral follicles as they became responsive to gonadotropins. In the mouse, after intensive GC proliferation and follicle growth, several follicles became dominant while others underwent atresia. Follicular atresia is a highly regulated process of programmed cell death, or apoptosis, particularly through local levels of FSH- mediated estradiol (E2) and androgen activity [51]. Atresia is generally initiated in GC, which at first undergo apoptosis through the activity of factors such as tumor necrosis factor (TNF) α or Fas ligand [51]. Furthermore, androgens were identified to play a prominent role in atresia, increasing the number of pyknotic GC and degenerated oocytes [75]. Novel mediators of atresia have been recently characterized as micro RNA (miR) 26b [76] or miR146b [77], shown to promote GC apoptosis in atretic follicles in pig ovaries [78]. Conversely, in dominant follicles, FSH activity was shown to elevate E2 levels through GC aromatase cytochrome P450 (CYP) 19A1 [50] with the upregulation of pro-survival growth factors: insulin-like growth factor (IGF) 1, epidermal growth factor (EGF), and basic fibroblast growth factor (FGF) [79]. Indeed, lack of FSH activity and low levels of E2 were associated with increased androgen activity, apoptosis, and atresia [80].

Estradiol is a predominant circulating estrogen in humans [81]. Its synthetic pathway starts in TC through the activation of the LH receptor and production of androgens from cholesterol [82]. After being transferred to GC, androgens are further converted into E2 by aromatase *CYP19A1*, a step regulated by FSH receptor activity [82]. As previously stated, the newly synthesized E2 plays a major role in dominant follicle selection [83]. Two nuclear E2 receptors (ER) have been characterized as auto-, paracrine mediators of E2 local actions, the ERα and the ERβ. Interestingly, the expression of ERs was shown to be controlled by gonadotropins [84], with ERβ being mainly evidenced in GC and ERα detected in TC and interstitial cells [85]. Studies with double knockout mice for ERα and ERβ showed the arrest of folliculogenesis at the secondary follicle stage [86]. Generally, after activation of its nuclear receptors, E2 binds to specific regions of the DNA, the estrogens response elements (EREs) [81], further regulating the transcription of genes controlling oocyte maturation and growth [84]. More recently, ERβ was shown to mediate the expression of *Junction Adhesion Molecule Like (Jaml)*, a gene needed for the communication between GC and oocyte, as well as other genes *Polypeptide N-Acetylgalactosaminyltransferase (Galnt) 6* or *Egf receptor (Egfr)*, necessary for preovulatory follicle development [87]. It is noteworthy that detailed E2 genomic actions in antral follicle development have been described elsewhere [88]. Nonetheless, E2 is also known to exert non-genomic actions through the membrane receptor, the G protein-coupled estrogen receptor (GPER) 1 [81]. E2 non-genomic effects were shown to involve the activation of intracellular signaling pathways, such as the Phospholipase C (PLC)/PKC pathway, the Ras/Raf/MAPK pathway, the PI3K/Akt kinase cascade, and the cAMP/protein kinase A (PKA) signaling pathway [81], which culminate with the activation of transcription factors from the STAT family, nuclear factor kappa B (NFκB), and CREB. Thus, E2 plays a prominent role in preovulatory follicle formation, activating multiple pathways and mediating growth and expansion of both somatic cells and oocyte.

Connexins are a family of proteins responsible for the organization of intercellular membrane channels of gap junctions, which allow for the transport of molecules, ions, and metabolites between GC and the oocyte. They were shown to be particularly important for preovulatory follicle formation. Indeed, the ovaries of connexin (Cx) 37 knockout mice showed follicular arrest at the preantral stage due to lack of bidirectional communication between GC and oocyte [89]. Another Cx, the Cx43, was also shown to be highly expressed in large antral follicles and mainly induced by E2 [90] and FSH [91]. Furthermore, a morphofunctional process determinant for follicular expansion and antrum formation is the establishment of microtubule TZPs between germ cell and somatic cells. The TZPs originate from a microtubule core in GC projected towards the oocyte, providing tracks for polarized translocation of secretory pathway organelles [92]. Studies on FSHβ knockout mice evidenced the retraction of TZPs from the oocyte, with drastic changes in oocyte chromatin remodeling and meiotic competence acquisition. Thus, coordinated actions of FSH and E2 on TZPs formation [93,94] ensure adequate communication between GC and oocyte during preantral to antral follicle transition.

Research in humans has recently revealed a number of important genes regulating preantral to antral follicle transition [30]. Importantly, *CX40* was identified as the most abundant Cx in human GC of antral and preovulatory follicles. Concerning oocyte-derived factors, *Activating Transcription Factor (ATF) 2* and *Eomesodermin (EOMES)* were abundantly expressed in the oocytes of preovulatory follicles, as well as *Bone Morphogenic Protein (BMP) 15* [30]. Finally, particular relevance was given to the NOTCH pathway during preovulatory follicle formation in women, with *Delta Like Canonical Notch Ligand (DLL)3* and *Jagged Canonical Notch Ligand (JAG) 2* being predominantly expressed in the oocyte and their receptors, *NOTCH2, NOTCH3*, and downstream target gene *Hes Family BHLH Transcription Factor (HES) 1*, highly expressed in GC. These findings highlight the role of the NOTCH signaling pathway regulating oocyte-mediated GC proliferation and differentiation [30].

To conclude, preovulatory follicle formation requires concerted action of gonadotropins FSH and LH on the crosstalk between GC and TC during steroidogenesis and E2 signaling. As a result, the formation of junctional complexes and TZPs create routes for the exchange of metabolites and signaling molecules between somatic cells and oocyte, determining follicle expansion and formation of the Graafian follicle.

### 5.2. Preovulatory Follicle Formation—The Role of Leptin

Leptin’s role in ovarian secretory activity is well established. In vitro studies with rat GC showed leptin treatment impaired E2 secretion [95], whereas the same effect was observed for human luteinized GC [96] or preovulatory follicles in swine [97]. Most importantly, experiments with GC collected from fertile woman showed that treatments with high doses of leptin had deleterious effects on FSH-mediated E2 secretion, mainly through IGF1 inhibition [98]. These results evidenced the detrimental effects of high leptin levels on E2 secretion by dominant follicles and consequent disruption of LH surge and impaired ovulation. Furthermore, a recent report has shown leptin interference with E2 secretion could be mediated by the induction of the neuropeptide cocaine- and amphetamine-regulated transcript (CART) at GC level [99]. Leptin negatively affected intracellular cAMP levels, MAPK signaling, and aromatase *Cyp19a1* mRNA expression with consequent downregulation of E2 synthesis [99]. Finally, we and others have also shown leptin effects on P4 synthesis, as leptin regulated, in a dose-dependent manner, the expression of cytochrome P450 side chain cleavage (P450 scc) and 3β hydroxysteroid dehydrogenase (HSD) [26,27]. Therefore, obesity and consequent hyperleptinemia can impair steroidogenesis in the growing antral follicle.

As previously stated, bidirectional communication between somatic cells and the gamete was revealed to be essential for oocyte maturation and follicular development progression. Interestingly, leptin was shown to modulate reorganization of actin, the main component of projections as TZPs, in systems like the hypothalamus [100] or nucleus pulposus cells in the intervertebral disks [101]. Recently, we have shown that pharmacological treatment of female mice with leptin altered the expression of genes associated with cytoskeleton organization in CC [6]. Moreover, the role of TZPs transferring leptin and *STAT3* from GC to oocytes has been previously reported [102]. Indeed, leptin was shown to regulate levels of Cx43 in the central nervous system (CNS) in vivo in mice [103], reiterating its potential role mediating intercellular communication between GC and the oocyte. On the other hand, the NOTCH pathway was shown by Zhang and co-workers to be upregulated in human preovulatory follicle [30]. Importantly, the crosstalk between leptin and NOTCH signaling was also shown in other systems, such as in vitro studies on glioblastoma cells [104]. Thus, disruption in ovarian leptin signaling during obesity can well affect steroidogenesis and the maintenance of channels for intercellular communication during preovulatory follicle formation.

### 5.3. Obesity and Leptin Signaling Disruption during Preovulatory Follicle Formation

Obesity has been strongly associated with increased circulating levels of both E2 and androgens, not only due to the ability of the adipose tissue to synthesize steroids [105] but also to low circulating sex hormone binding globulin levels and suppression of gonadotropin release. Moreover, ovarian TC are known to respond to insulin during androgen synthesis [1]. Seminal work by Wu and co-workers clearly showed the link between hyperinsulinemia in DIO mice and increased ovarian androgen production through hyperactivation of CYP17 at TC level [106]. On the other hand, Souter et al. also correlated gain in body mass index (BMI) with E2 secretion by preovulatory follicles in obese women [107]. Finally, studies in rats evidenced the link between obesity and the lack of preovulatory surge of P4 and LH [108]. Hence, obesity clearly disrupts steroidogenesis, causing a predisposition to premature follicular atresia and anovulation.

**Figure 5:**
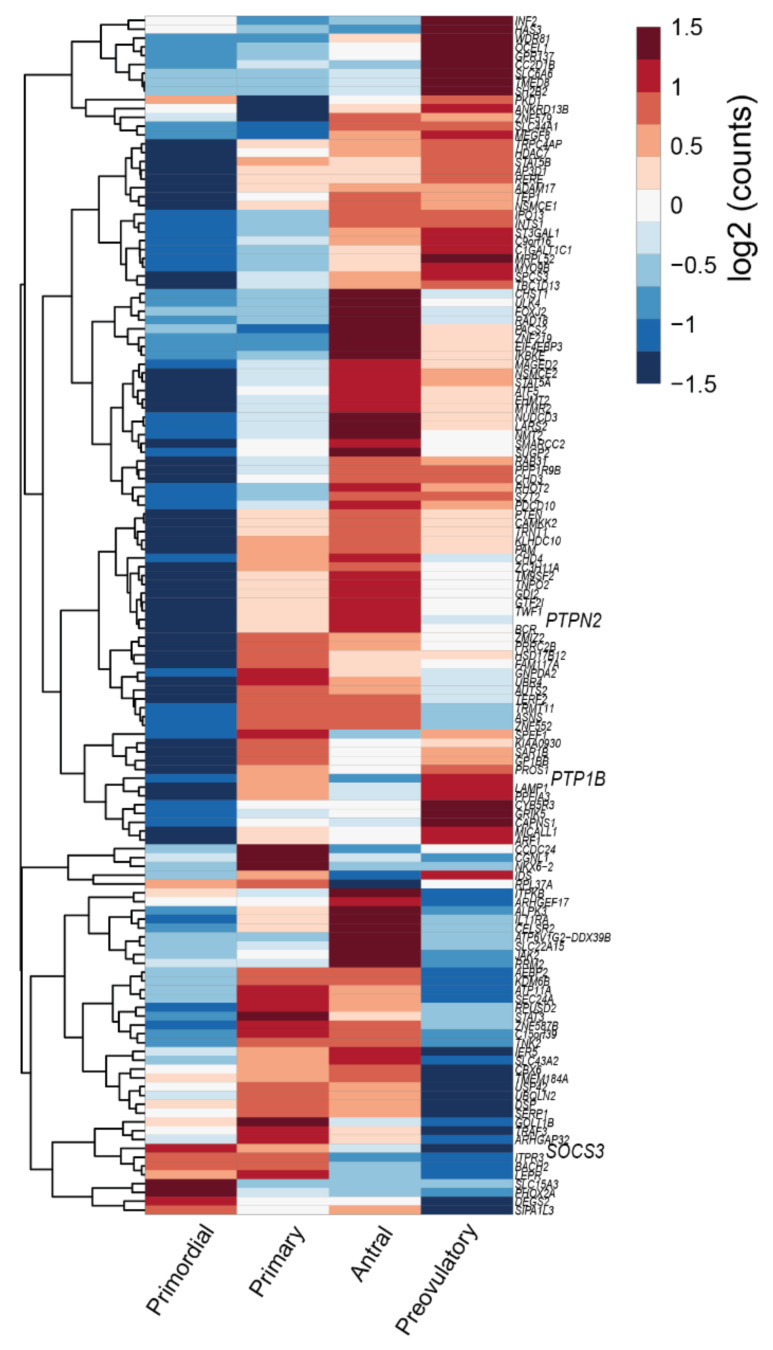
Heatmap representing transcription profile of genes identified as differentially expressed in cumulus cells of mice both fed high fat diet for 4 wk and treated with leptin (for details please see [6]) in human granulosa cells throughout folliculogenesis. Primordial = primordial follicle; Primary = primary follicle; Antral = antral follicle; Preovulatory = preovulatory follicle. Color code from blue to red indicates the relative gene expression level from low to high, respectively. Data from Zhang et al. 2018 [30]; 134 DEGs plus 10 leptin pathway genes—Table S2. Suppressor of cytokine signaling (SOCS) 3, protein tyrosine phosphatase non-receptor type 2 (*PTPN2*), protein tyrosine phosphatase (*PTP*) 1B.

The negative impact of obesity on preovulatory follicle formation can be also related to the expression of leptin signaling inhibitors *PTP1B* and *SOCS3*. Indeed, dephosphorylation of human estrogen receptor by *PTP1B* was shown to reduce E2 binding capacity [109]. In breast cancer cells, *PTP1B* was also shown to reduce aromatase activity when overexpressed [110]. Therefore, during leptin resistance, potential increased *PTP1B* activity [111] could impair aromatase activity and E2 levels in the follicle. Conversely, *SOCS3* was previously shown to block ERK1/2 signaling, an important kinase in the LH-mediated oocyte resumption of meiosis, ovulation, and luteinization [112]. Finally, we asked to what extent common DEGs previously identified in CCs collected from both leptin treated and early obesity mouse protocols (4 wk HFD) could be already expressed in GC from antral and preovulatory follicles. We used once more the data from human GC transcriptome throughout folliculogenesis [30] and plotted the DEGs from our study in mice (134 DEGs plus 10 leptin pathway genes—Table S2) [6]. Interestingly, most of the genes were actively transcribed in GC from human antral and preovulatory follicles (Figure 5) as *PTPN2* and *PTP1B*, suggesting this way that previously observed changes in gene expression in CC in early obesity, driven by increased leptin activity, could be seemingly important for GC from preantral and antral follicles. To conclude, dysregulation of leptin signaling inhibitors, through hyperactivation of ObRb and activity of downstream leptin genes, can equally affect preovulatory follicle formation in obese mothers.

## 6. Oocyte Maturation: The Last Step before Fertilization

The acquisition of developmental competence by the oocyte is a prerequisite for fertilization and sustained embryo development. Oocyte maturation involves nuclear and cytoplasmic transformation, which largely determine the quality of the female gamete. Oocyte meiotic division remains blocked until hormonal signals unlock the process. The nuclear transformation encompasses dramatic changes in chromatin organization, which evolves from a decondensed and transcriptionally active state in preantral follicles with a non-surrounded nucleolus (NSN) oocyte [113] into condensed and silenced chromatin in antral follicles with a surrounded nucleolus (SN) oocyte [113]. The cytoplasmic maturation culminates with the reorganization of organelles and preparation for completion of meiosis. After fertilization, meiosis is completed and maternal transcripts are degraded, with the zygote becoming transcriptionally independent and undergoing intense cell division. In the present section, we describe the signaling pathways leading major events orchestrating oocyte maturation and early embryo development, particularly, highlighting potential links to leptin signaling and its disruption during obesity.

### 6.1. Regulation of Oocyte Maturation

Oocyte nuclear maturation is key for meiosis accomplishment and fertilization [113]. Both human and mouse oocytes from preovulatory follicles are capable of re-entering meiosis; nonetheless, they remain arrested in prophase I until exiting the follicle. This allows for the accumulation of transcripts and proteins, chromatin reorganization, and cytoplasm maturation. Meiotic arrest was shown to be sustained by high cAMP concentrations in the oocyte through the activity of constitutive G protein coupled receptor (GPR) 3 and/or GPR 12 [114]. Hence, cAMP was shown to ensure high levels of PKA activity, leading to inactivation of cell division cycle 25 homolog B (CDC25B) and maturation promoting factor (MPF) and, therefore, enforcing meiotic arrest [115]. Receptors GPR3/12 can be activated by multiple lysophospholipids, such as sphingosylphosphorylcholine (SPC) and sphingosine 1-phosphate (S1P), which were shown to delay spontaneous oocyte maturation [116]. Moreover, cAMP degradation by phosphodiesterase 3A (PDE3A) in the oocyte was also shown to be prevented by exogenous cyclic guanosine monophosphate (cGMP), which appears to be transferred from GC via gap junctions in both mice [117] and humans [118]. The GC-derived cGMP is generated by guanylyl cyclase activity after activation of natriuretic peptide receptors (NPR) by their ligands natriuretic peptides (NPP). The predominant receptor in GC is NPR2 with its cognate ligand NPPC [116]. Interestingly, the expression of the NPR2/NPPC system was shown to be regulated by E2 signaling [84]. Another recent study showed that transforming growth factor (TGF) β upregulated the expression of NPPC [119]. Furthermore, sex steroids were linked to meiosis resumption in a transcription independent manner, with testosterone shown to induce oocyte maturation [120]. After the LH surge, oocyte nuclear maturation and meiosis resumption started with germinal vesicle (GV) breakdown (BD), morphologically characterized by the dissolution of the oocyte nuclear envelope. Indeed, LH was shown to decrease cGMP levels through suppression of NPR2 activity and inhibition of NPPC transcription in GC [116]. Finally, after LH surge, cGMP production was stopped, Cx43 phosphorylated and gap junctions closed, and PDE3A activated in the oocyte with rapid drop in cAMP levels [116]. As a result, MPF could be activated and meiosis resumed.

Another pivotal step during oocyte maturation is the chromosome condensation, generally followed by meiosis progression through prometaphase I, metaphase I, anaphase I, and extrusion of first polar body (PB) in telophase I. Finally, adequate microtubule polymerization is also determinant for oocyte maturation and a prerequisite for correct spindle assembly and chromosome trafficking. The process of microtubule nucleation and subsequent spindle assembly is considered chromosome-dependent in human oocytes [121]. In contrast, in mice, the spindle formation was shown to be driven by the organization of microtubule organizing centers (MTOCs). Once the first PB is extruded, oocyte reassembles the MII spindle with chromosomes aligned in equatorial plate, ready to extrude a second PB upon fertilization.

Cytoplasmic oocyte maturation starts during the GVBD and comprises reorganization of the cytoplasm, remodeling and repositioning of intracellular organelles, movement of vesicles like Golgi and EnR, but also mitochondria repositioning around spindle [122]. Oocytes from most species, including humans, contain Balbiani bodies, which are unique transient cytoplasmic structures for RNA storage and control of translation [123]. Additionally, rodent oocytes contain also cytoplasmic lattices to store ribosomes. Studies with transgenic mice showed the involvement of peptidyl arginine deiminase 6 (PADI6) [124] in the organization of such structures, as well as the maternal effect gene maternal antigen that embryos require (Mater) [125]. Indeed, during oocyte maturation, a tightly regulated program of maternal mRNA synthesis, degradation, and storage, particularly mRNAs related to ribosome and mitochondria biogenesis, is determinant for acquisition of developmental competence and early embryo development before zygotic genome activation.

The three previously described steps for oocyte maturation (oocyte nuclear maturation and meiosis resumption, chromatin condensation, and cytoplasm maturation) are entirely dependent on oocyte metabolism. It provides energy for meiotic progression, balancing intracellular redox and osmotic potential, and provides building blocks for oocyte growth [122]. Indeed, it was shown that oocytes collected from prepubertal cows had reduced ability to generate viable embryos due to altered metabolic profile with regard to adult animals [126]. Furthermore, genes controlling metabolism were shown to be upregulated in mouse MII oocytes compared to GV oocyte [127]; also, the expression of metabolic genes differed between in vitro and in vivo maturation in bovine oocytes [128]. Mouse oocytes mainly use pyruvate as an energy source for oxidative phosphorylation as pyruvate can exclusively support maturation, maintain viability, and promote cleavage of zygote in the absence of CC [129]. Glucose is mostly metabolized by CC, and together with glucose-6-phosphate, lactate or pyruvate can be transported into the oocyte via gap junctions. Two main oocyte- secreted factors, BMP15 and GDF9, were shown to control CC metabolism [130]. Furthermore, mitochondria significantly contribute to cellular metabolism, and deficiencies in aerobic mitochondrial metabolism have been linked to impaired oocyte competence [131]. Finally, lipid metabolism also plays a role in energy production in oocyte and embryo in mice, supporting its maturation and development. In response to LH surge, fatty acid oxidation takes place in the oocyte, with long chain fatty acid metabolism being a source of acetyl coenzyme A (acyl-CoA), which enters tricarboxylic cycle and electrons for nicotinamide adenine dinucleotide phosphate (NADPH) production in the electron transport chain for adenosine triphosphate (ATP) production [132]. On the other hand, leptin is known to promote lipid oxidation and regulation of triglyceride cellular homeostasis [133]. Therefore, changes in leptin levels can affect oocyte fatty acid oxidation and energy provision during oocyte maturation.

In conclusion, major steps for oocyte maturation encompass dramatic structural changes at the nuclear and cytoplasmic level, which rely on the metabolic performance of both oocyte and CC. Indeed, oocyte quality is known to affect embryo development, being, therefore, the integrity of oocyte maturation and metabolic performance determinant for early embryo development and implantation.

### 6.2. Leptin Effects on Oocyte Maturation

Leptin involvement in oocyte maturation and early embryo development was previously documented. Intriguingly, leptin was shown to support meiotic progression and developmental competence of bovine oocytes, as well as fertilization and blastocyst development [134]. Furthermore, leptin promoted nuclear and cytoplasmic maturation via MAPK pathway activation in porcine oocytes, as in vitro studies showed increased proportion of oocytes reaching MII stage, upregulation of cyclin B1 protein expression, and enhanced embryo development [135]. In another in vitro study, the supportive effects of leptin on GVBD formation in oocytes from preovulatory follicles were demonstrated, enhancing first PB extrusion and development of preimplantation embryos, mainly through MEK1/2 signaling [136]. Reports in pancreatic beta cells revealed leptin’s ability to stimulate adenylate cyclase and increase cAMP cellular levels [137] and activate PDE3B [138]. Moreover, leptin treatment increased intracellular cAMP level and PKA activity in murine macrophages [139]. Indeed, in different cellular contexts, leptin was also shown to modulate factors, such as cAMP and PKA activity, known to have a prominent role in meiosis reactivation and maturation of the gamete. Finally, the role of leptin on glucose homeostasis systemically is well described through the modulation of the IRS/PI3K pathway [140]. Leptin was also shown to attenuate the deleterious impact of high glucose levels in oocyte, promoting glycolysis and oocyte maturation [141]. Thus, changes in leptin signaling during obesity can produce expected changes in oocyte metabolism.

### 6.3. Oocyte Maturation and Early Embryo Development in Obese Mothers

Obesity leads to severe systemic hormonal imbalance with drastic consequences for oocyte maturation and embryo development. Firstly, maternal obesity was shown to alter insulin, glucose, and free fatty acid concentration in follicular fluid, directly affecting oocyte metabolism and reducing oocyte maturation [142]. Secondly, insulin-stimulated glucose uptake was shown to be impaired in CC isolated from mice treated with HFD, suggesting the establishment of insulin-resistance [143]. Indeed, activation of the polyol pathway during hyperglycemia was shown to negatively affect metabolism and CC–oocyte communication [144]. As a result, in obese mothers, oocyte maturation, fertilization rate, and embryo quality were significantly decreased [145]. Moreover, oocytes derived from obese mice after in vitro fertilization and culture presented reduced development [145]. Furthermore, studies in mice clearly linked maternal obesity with oocyte and zygote increased mitochondrial potential, mitochondrial DNA content and biogenesis, and generation of reactive oxygen species (ROS) [2]. Importantly, the absence of mitophagy was presented as the main cause of mitochondrial dysfunction in oocytes and early embryos from obese mothers [12]. Another recent report also linked lack of expression of Stella in oocytes from obese mice with increased hydromethylation in the zygote and DNA instability [5]. Thus, obesity can drastically affect both oocyte and embryo quality. Finally, with regard to direct effects of altered leptin signaling components in oocyte maturation, *SOCS3* mRNA was shown to be decreased in human CC collected from patients with polycystic ovary syndrome (PCOS) [146]. Indeed, *SOCS3* was suggested as a biomarker of oocyte or embryo competence in CC of PCOS patients. In summary, maternal obesity leads to disruption in mitochondrial dynamics, excessive free fatty acids, and cellular damage, which affect not only the oocyte but may also hamper embryo development.

## 7. Conclusions

**Figure 6:**
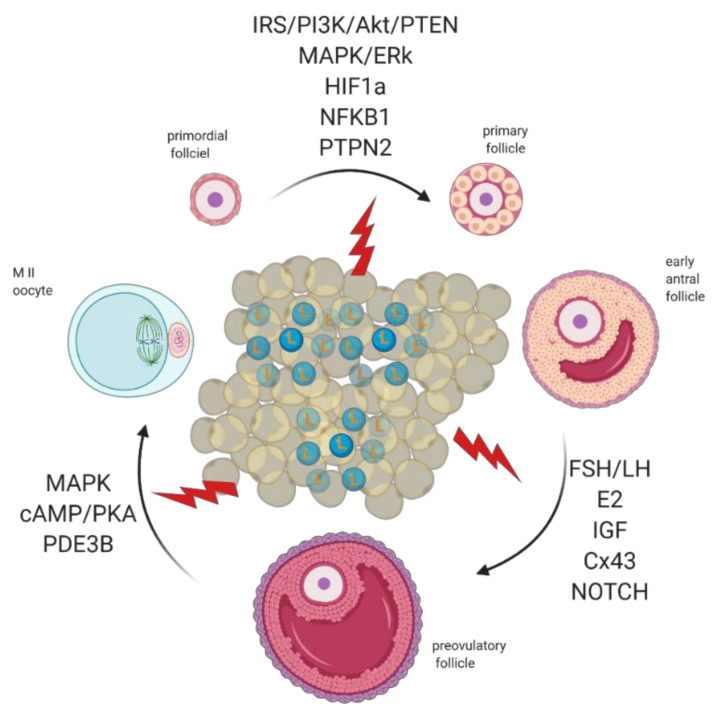
Adipose tissue secretes excessive amounts of leptin (L) during obesity. Leptin signaling in the ovaries of obese mothers is altered, culminating with the establishment of leptin resistance. As a result, signaling pathways governing primordial follicle activation, preovulatory follicle formation, and oocyte maturation can be affected. Created with BioRender.com, accessed on 1 November 2020.

Folliculogenesis regulation is a complex process that depends on the crosstalk between local and systemic factors, accounting, therefore, for its vulnerability to maternal physiological fitness. Indeed, leptin, an established local regulator of folliculogenesis, presents increased circulating levels in obese mothers with major consequences for follicular activation, recruitment, and growth. In addition to increased ovarian ObRb activation and altered leptin signaling, deleterious effects of systemic hyperleptinemia during obesity can also result from local overexpression of mediators of leptin resistance, such as *SOCS3*. As a result, leptin’s prominent role as suppressor of follicular pool activation can be affected in obese mothers, with consequent POF. During preovulatory follicle formation, altered leptin signaling affects not only steroidogenesis but the communication between GC and oocyte, which is known to be key for antrum formation and oocyte growth. Finally, changes in leptin signaling and impaired metabolism may also incur drastic consequences for oocyte maturation, hampering meiosis resumption and cytoplasmic maturation (Figure 6). Hence, advances in our understanding of the role of leptin on ovarian pathophysiology during obesity should unravel innovative tools to monitor the quality of the oocyte during disease progression, potentially preventing pregnancy failure and ensuring the birth of a healthy offspring.

## Data Availability

The data presented in this study are available on request from the corresponding author.

## Supplementary Materials

The following are available online at https://www.mdpi.com/article/10.3390/ijms22084270/s1, Table S1: Summary of the studies cited in the present review, regarding species, nature of study (in vivo or in vitro), type of report (basic study, clinical report, review/book chapter); Table S2: List of differentially expressed genes (A) The overlap between differentially expressed genes in cumulus cells collected from mice after 4 weeks of diet-induced obesity or 16 days of leptin treatment [6]. (B) The list of components of leptin signaling pathway.

## Abbreviations

acyl-CoA	acetyl coenzyme A
Akt	Protein kinase B
AMH	Anti-Mullerian hormone
ATF	Activating transcription factor
ATP	Adenosine triphosphate
BDNF	Brain-derived nerve factor
BMI	Body mass index
BMP	Bone morphogenic protein
cAMP	Cyclic adenosine monophosphate
CART	Cocaine- and amphetamine-regulated transcript
CC	Cumulus cells
CDC25B	Cell division cycle 25 homolog B
cGMP	Cyclic guanosine monophosphate
CNS	Central nervous system
COCs	Cumulus–oocyte complexes
CREB	Activates cyclic AMP-response element binding protein
Cx	Connexin
CYP	Cytochrome P450
DEGs	Differently expressed genes
DIO	Diet-induced obesity
DLL	Delta like canonical notch ligand
dpc	Days post coitum
E2	Estradiol
EGF	Epidermal growth factor
Egfr	Epidermal growth factor receptor
EOMES	Eomesodermin
EnR	Endoplasmic reticulum
ER	Estradiol receptor
EREs	Estrogens response elements
ERK	Extracellular signal regulated kinase
FGF	Basic fibroblast growth factor
FIGa	Folliculogenesis-specific basic helix–loop–helix
FOXO	Forkhead box O
FSH	Follicule-stimulating hormone
Galnt	Polypeptide N-acetylgalactosaminyltransferase
GC	Granulosa cells
GDF	Growth differentiation factor
GnRH	Gonadotropin releasing hormone
GPER	G protein coupled Estrogen receptor
GPR	G protein coupled receptor
GVBD	Germinal vesicle breakdown
HES	Hes family BHLH transcription factor
HFD	High-fat diet
HSD	Hydroxysteroid dehydrogenase
IGF	Insulin-like growth factor
IRS	Insulin receptor substrate
JAG2	Jagged canonical notch ligand
JAK	Janus kinase
Jaml	Junction adhesion molecule like
Kit	Stem cell factor receptor
LH	Luteinizing hormone
Lhx8	LIM homeobox protein
MAPK	Mitogen-activated protein kinase
Mater	Maternal antigen that embryos require
MEK	Mitogen-activated protein kinas
MII	Metaphase II oocyte
miR	Micro RNA
MPF	Maturation promoting factor
MTOCs	Microtubule organizing centers
mTOR	Mammalian target of rapamycin
mTORC1	Mammalian target of rapamycin complex 1
NADPH	Nicotinamide adenine dinucleotide phosphate
NFKB	Nuclear factor kappa B
NPP	Natriuretic peptides
NPR	Natriuretic peptide receptors
NSN	Non-surrounded nucleolus oocyte
NT	Neurotrophin
ObR	Leptin receptor
ObRb	Leptin receptor isoform b
p27	Cyclin-dependent kinase inhibitor 1B
P4	Progesterone
P450scc	Cytochrome P450 side chain cleavage
PADI	Peptidyl arginine deiminase
PB	Polar body
PCOS	Polycystic ovary syndrome
PDE3A	Phosphodiesterase 3A
PDK	Phosphoinositide-dependent kinase
PGC	Primordial germ cells
PI3K	Phosphatidylinositol 3 kinase
PIP2	Phosphatidylinositol-4,5-biphosphate
PIP3	Phosphatidylinositol-3,4,5-triphosphate
PKA	Protein kinase A
PKC	Protein kinase C
PLC	Phospholipase C
POF	Premature ovarian failure
PTEN	Phosphatase and tensin homolog
PTP1B	Protein tyrosine phosphatase
PTPN2	Protein tyrosine phosphatase non-receptor type 2
RNA	Ribonucleic acid
ROS	Reactive oxygen species
rpS6	Ribosomal protein S6
S1P	Sphingosine 1-phosphate
S6K1	Ribosomal protein S6 kinase beta-1
SCF	Stem cell factor
SHP-2	SH2-domain containing protein tyrosine phosphatase
SIRT	Sirtuin
SN	Surrounded nucleolus oocyte
SOCS3	Suppressor of cytokine signaling 3
SPC	Sphingosylphosphorylcholine
STAT	Signal transducer and activator of transcription
TC	Theca cells
TGF	Transforming growth factor
TNF	Tumor necrosis factor
TSC	Tuberous sclerosis complex
TZPs	Transzonal projections
wk	Weeks
